# A preliminary evaluation of the reliability of a modified functional scoring system for assessing neurologic function in ambulatory thoracolumbar myelopathy dogs

**DOI:** 10.1186/s12917-015-0557-8

**Published:** 2015-09-24

**Authors:** Chung-Sheng Lee, R. Timothy Bentley, Hsin-Yi Weng, Gert J. Breur

**Affiliations:** Department of Veterinary Clinical Sciences, College of Veterinary Medicine, Purdue University, 625 Harrison Street, West Lafayette, IN 47907 USA; Present Address: Department of Clinical Sciences, College of Veterinary Medicine, Mississippi State University, Starkville, MS 39762 USA; Department of Comparative Pathobiology, College of Veterinary Medicine, Purdue University, 625 Harrison Street, West Lafayette, IN 47907 USA

**Keywords:** Canine, Gait analysis, Spinal, Intervertebral disc disease, Outcome

## Abstract

**Background:**

The objective of this study was to develop and assess the reliability of a modified scoring system for evaluating the function of the two pelvic limbs separately, in ambulatory thoracolumbar myelopathy dogs. A previously established neurologic score scale for dogs with T3-L3 lesions was modified in order to provide a separate score for each pelvic limb.

**Results:**

Seventeen ambulatory dogs with thoracolumbar myelopathies were evaluated. Using the new scale, two observers independently performed 22 observational gait analyses (OGAs) in ten dogs without videotape. Another 18 OGAs were performed in seven dogs by watching videotapes of them ambulating. There was poor agreement (concordance correlation coefficient, 0.87) between the two observers for all 40 OGAs. When stratified, the agreement was moderate (concordance correlation coefficient, 0.90) in the OGAs without videotaping and poor (concordance correlation coefficient, 0.80) for the OGAs based on videotapes. For the decision regarding which pelvic limb was more severely affected, a fair agreement (kappa value, 0.30) between the two observers was noted. Without videotape there was only slight agreement (kappa value, 0.05), but with videotape there was moderate agreement (kappa value, 0.56).

**Conclusions:**

The modified scoring system in this study provides moderate reliability in assessing the functional neurologic status of each pelvic limb, by OGA without videotape, in canine T3-L3 patients. Further development of this scoring system is required. However, imperfect agreement when visually quantifying neurological deficits is not unexpected.

## Background

Various behavior analyses have been used in animals with myelopathies. These methods include spinal reflexes, postural responses, locomotor and sensory function tests, conditioned behavior evaluations, endpoint measurements, and kinematic, kinetic and electrophysiological measurements. These have been applied to animals with spinal cord injuries in both experimental studies and clinical trials in order to evaluate functional recovery [1–5]. Canine gait analysis has been defined as a systematic process of measuring gait patterns that enables clinicians and researchers to accurately and efficiently explore the gait cycle [6, 7]. Gait analysis allows evaluation of limb function and can be classified as observational gait analysis (OGA) or instrumented gait analysis (IGA) [8]. In dogs with spinal cord injury, OGA can be used to detect ataxia including limb-crossing, dragging of limbs, reduced speed, wide-based stance, difficulty with flexing joints, irregular steps and a tendency for higher impacts of the limbs when hitting the ground [9]. Paresis, paralysis and spasticity can also be discerned. To improve objectivity, several semi-quantitative methods of OGA have been developed to evaluate the neurologic function of animals following spinal cord injury [6, 10–16]. The most commonly used semi-quantitative method of OGA in canine myelopathies is the modified Frankel score [6, 10–12], which was initially developed for humans with traumatic myelopathy [17]. Recently, the reliability of some of these semi-quantitative methods has been tested including the functional scoring system of Olby [13], the Texas spinal cord injury score [14] and gait scoring when walking on a treadmill [15].

In IGA, equipment such as a force plate or pressure sensing walkway is used to collect, store, analyze and report quantitative gait variables of individual limbs [18]. Instrumented gait analysis is an objective way to evaluate canine gait and can provide information on recovery as well as aid with diagnosis or treatment planning. Recently, several studies on the use of kinetic and kinematic gait analysis in dogs with neurological disease have been reported [19–28]. These studies were limited by dog weight and breed and thus their results might not be generalizable to the overall dog population. The IGA results of these studies were also not compared to OGA scores, and IGA provides data for each individual thoracic and pelvic limb while current OGA scoring would only give a single score.

In this study, we modified the OGA scoring system of Olby et al. [13] in order to evaluate the function of each pelvic limb separately in ambulatory thoracolumbar myelopathy dogs. We report the inter-rater agreement when this new OGA scoring system is applied by two different investigators. Our larger goal is to develop an OGA scoring system for individual pelvic limbs that can be used to establish and evaluate IGA in dogs of all body weights and breeds with a T3-L3 myelopathy.

## Methods

Ambulatory paraparetic dogs were evaluated by the Neurology service at Purdue University Veterinary Teaching Hospital for myelopathy during new or recheck appointments. OGAs were performed by two observers, one veterinary neurology resident (observer 1) and one board-certified veterinary neurologist (observer 2). The functional scoring scale used in this study (Table 1) was modified from a scoring method developed by Olby et al. [13] in order to evaluate each pelvic limb separately. In addition to giving each pelvic limb a separate score, if giving the two limbs equal scores, the observer was instructed to specify which limb was worse.

**Table 1:** The modified functional scoring system used in this study

Each pelvic limb is evaluated individually
1. Non-ambulatory (scores 2–10 indicate the dog is ambulatory and can take at least 4 steps)
2. Weight-bearing protraction of the pelvic limb <10 % of the time
3. Weight-bearing protraction of the pelvic limb 10 to 50 % of the time
4. Weight-bearing protraction of the pelvic limb >50 % of the time
5. Weight-bearing protraction 100 % of the time with reduced strength of the pelvic limb; mistakes >90 % of steps (e.g., scuffing foot on protraction, standing on dorsum of foot)
6. Weight-bearing protraction of the pelvic limb 100 % of the time with reduced strength; mistakes 50 to 90 % of steps
7. Weight-bearing protraction of the pelvic limb 100 % of the time with reduced strength; mistakes <50 % of steps
8. Ataxic pelvic limb gait with normal strength (including ability to rise from a sitting down position); mistakes >90 % of steps (e.g., crossing of pelvic limbs, skipping steps, bunny-hopping, scuffing foot on protraction)
9. Ataxic pelvic limb gait with normal strength; mistakes 50–90 % of steps
10. Almost normal pelvic limb gait with normal strength; mistakes <50 % of steps

To avoid the risk of further injury during the gait analysis, any history of trauma or possible vertebral instability was revealed to observer one by support staff before performing OGAs. These cases were excluded from the study. Both observers were otherwise blinded to all other clinical information prior to performing OGA. Neurologic examination was completed after performing the OGA or after videotaping the gait for future analysis (see below). The inclusion criterion was a clinical diagnosis of a T3-L3 myelopathy based on neurological examination; patients were excluded if the lesion was subsequently localized to L4-S3 or anywhere else by neurologic examination. Patients could be evaluated multiple times during subsequent hospital visits (at least 2 weeks apart). The results of any diagnostic procedures such as advanced imaging or surgical biopsy were also reviewed for this study.

In the first phase of the study, OGAs were performed by observing the patient walking on a non-slip surface. Each observer was allowed to watch the patient walk until certain of the appropriate score; all dogs were allowed to take a rest for 10 min if they showed any signs of fatigue. The OGAs by the two observers were separated by at least 10 min. In the second phase, videotape was obtained from both sides, in front and behind when the patient was walking on the wooden walkway in the Purdue University Animal Gait Analysis Laboratory. Videotaping was continued until good footage was achieved, that is to say the patient walked at a constant speed, without veering to one side, pulling at the leash, trying to sit down, etc. Each observer separately evaluated the same video, and was allowed to pause and playback the video until certain of the appropriate score. Videos were analyzed in batches by each investigator. All clients provided written consent to gait analysis and their dogs being videotaped. The study was approved by the Purdue Animal Care and Use Committee.

In preparation for the study, five ambulatory paraparetic dogs were evaluated in order to allow the observers to practice the utilization of the new OGA scoring system. The two investigators scored these dogs separately, and then discussed their results in order to clarify definitions and the implementation of the scoring system. The scores obtained during this preparatory training phase were not used in the statistical analysis.

### Statistical analyses

Descriptive statistics were reported as median scores and the range of scores. The concordance correlation coefficient [29, 30] was used to assess the inter-rater reliability of the scoring method. A concordance correlation coefficient of <0.90 is considered poor, 0.90–0.95 moderate, 0.95–0.99 substantial, and >0.99 almost perfect [30]. The kappa statistic [31, 32] was utilized to evaluate the agreement upon the limb with the lower score (i.e., left versus right pelvic limb versus both limbs equal) between the two observers. A kappa value of <0.20 indicates slight agreement, 0.21–0.40 fair agreement, 0.41–0.60 moderate agreement, 0.61–0.80 substantial agreement, and >0.81 almost perfect agreement [32].

## Results

A total of 17 dogs were enrolled in the study between April 2013 and July 2014. OGAs were performed on 16 dogs during recheck appointments and only one dog was evaluated during a new appointment. One dog was evaluated at two different time points during recovery and another three times, providing 20 OGAs (40 limbs) per observer or 40 OGAs (80 limbs) between the two observers. The seventeen dogs included Dachshund (*n* = 8), mixed breed (*n* = 2) and Coton de Tulear, Maltese terrier, American pitbull terrier, Pug, Basset Hound, Pekingese and Scottish terrier (*n* = 1 of each). The median age was 7.9 (range, 3.2–13.8) years old. There were three intact male dogs, four castrated male dogs, one intact female dog and nine spayed female dogs. The median body weight was 8.1 (range, 4.0–21.2) kg.

Surgical biopsy, computed tomography or magnetic resonance imaging had been previously performed in 15 dogs. Intervertebral disk disease was diagnosed in 14 dogs by computed tomography/magnetic resonance imaging with (*n* = 13) or without (*n* = 1) surgery. Meningioma (*n* = 1) was diagnosed by surgical biopsy. In two dogs, intervertebral disc disease was diagnosed by signalment, history and neurologic examination only.

The first 11 evaluations were performed by visual observation and the next nine evaluations by reviewing a standardized video recording. Table 2 summaries the median and range scores of the OGAs.

**Table 2:** Median scores and range of the OGAs in two observers with and without videotape

	All OGAs		OGAs without videotape		OGAs with videotape	
	(*n* = 40)		(*n* = 22)		(*n* = 18)	
Observer	1	2	1	2	1	2
Median score (range)	9 (3–10)	9 (4–10)	9 (3–10)	9.5 (4–10)	8 (5–10)	9 (5–10)

In the training phase with the first five dogs, agreement was initially variable, but minor changes were made to the scoring system (see definitions in Table 1 compared to Olby et al. [14]) and agreement was then subjectively good.

### Inter-rater reliability of the scoring method

**Fig. 1:**
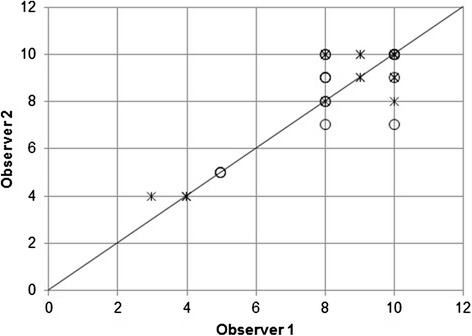
Scatterplot of the scores of pelvic limbs in observer 1 against observer 2. The scores were acquired from the evaluation of right and left pelvic limbs individually in 40 OGAs. Asterisk (_*_) denotes the score of 22 OGAs without videotape and circle (O) denotes the scores of 18 OGAs with videotape

Of the 40 limbs (20 pairs of limbs) evaluated, the observers agreed on the score for 22 limbs (55 %), disagreed by one point on 11 limbs (27.5 %), disagreed by two points on six limbs (15 %) and disagreed by three points on one limb (2.5 %). Figure 1 shows the OGA scores by the two observers with the 45 degree diagonal line denoting a perfect agreement. The concordance correlation coefficient between the two observers for all 20 cases was 0.87 (95 % confidence interval, 0.77–0.93). For the 11 cases without videotape, the observers had 55 % (12 of 22 limbs) agreement with a concordance correlation coefficient of 0.90 (95 % confidence interval, 0.78 to 0.96). For the nine cases analyzed with videotape, the observers had 56 % (10 of 18 limbs) agreement with a concordance correlation coefficient of 0.80 (95 % confidence interval, 0.55 to 0.92).

### Inter-rater agreement on the limb with the lowest score

Regarding the limb with the lower score (left vs. right vs. equal), agreement between the two observers was 65 % (13 of 20 cases) and the kappa value was 0.30. For the analyses without videotapes agreement was 55 % (six of 11 cases) and the kappa value was 0.05. For the analyses with videotape agreement was 78 % (seven of nine cases) and the kappa value was 0.56.

When giving both pelvic limbs the same score, the observers were asked to state which limb was subjectively worse. In 11 of 20 cases (55 %), the observers agreed on the worse limb. In the remaining nine cases (45 %) in which there was disagreement, the neurological dysfunction was relatively symmetrical, with the two limbs being given equal scores by one (*n* = 3) or both (*n* = 6) investigators; disagreement occurred only when at least one observer gave the limbs equal scores and had to give a subjective opinion as to which limb was worse.

## Discussion

The results of this preliminary study suggest that the evaluated scoring system had a weak inter-observer agreement regarding the score of each pelvic limb (concordance correlation coefficient, 0.87) and a fair agreement regarding the more severely affected limb (kappa value, 0.30) with an inconclusive effect of using videotape on OGA scoring.

Many invasive or histologic methods have previously been established to quantify the degree of injury after acute spinal cord injury [10, 11, 33–46]. Among reported methods, only OGA and IGA evaluate limb function. Of these two techniques, OGA is cheaper, convenient, non-invasive, repeatable and does not require any additional equipment. However, IGA is considered to be more objective [7].

In the modified Frankel system and the Texas spinal cord injury score [14], each limb can be evaluated individually. Utilizing the Texas spinal cord injury score, seven different gait grades could be applied to each limb but ambulatory patients will fall into only three different grades including normal. The score with the modified Frankel system has only two different ambulatory grades of spinal injury (i.e. normal gait with spinal hyperesthesia or ambulatory paraparesis). The 14-point functional scoring system of Olby [13] was established to evaluate small functional differences in dogs with acute spinal cord injury. When applied only to ambulatory dogs, scores fall in a nine point scale from a low grade of weight bearing to normal pelvic limb gait, allowing quantification of many different levels of ambulatory paraparesis-ataxia. This scoring system therefore had many of the characteristics that we desired, including high levels of agreement between two investigators, and was chosen as the basis on which to develop our scoring system. However, we required a scoring system that provided data on each pelvic limb separately and the Olby system was designed to provide a single score for each dog. Having modified the Olby system for our purposes we believed it was important to assess the interobserver agreement of the new scoring system. This included specifically evaluating the agreement regarding a very subjective decision in many cases, namely which limb was given the lower score. A 10-point score was described in this system and scores for each pelvic limb will fall within nine grades (grades two through ten) if the dog is ambulatory. Our scoring system also necessitated that the observer state which limb they considered most abnormal, whenever both pelvic limbs were given the same score. The goal of this design was to increase the ability to quantify a subtle difference of neurologic function in the pelvic limbs. It is needed since we expect to use this scoring system to evaluate the practicability of IGA in spinal cord injured dogs by comparing the correlation between IGA and OGA.

The validity and reliability of newly developed scoring systems for spinal cord injury patients have been evaluated in different ways in previous studies. Experimentally, the validity of Basso, Beattie, Bresnahan locomotor rating scale was evaluated by its association with histopathology in rat spinal cords [47]. Clinically, the validity of scoring systems has been evaluated by comparing to another well-accepted system or examining serial scores which correspond to the change of neurological function [48–53] in human medicine. The reliability of scoring systems has been tested by determining the inter-rater agreement in both human and veterinary medicine [6, 16, 17, 54, 55].

In this study, we assessed the reliability of our new scoring system by evaluating inter-rater agreement. The poor to moderate inter-rater agreement was not unexpected as visual scoring systems are subjective. Observers are required to implement the scoring system the same way each time, even when time has passed since the preceding patient was evaluated. Another way to evaluate reliability is to assess intra-observer agreement. However, this would have been very limited in our study, as over half the patients were not videotaped.

In our study, OGA based on videotaping trended towards a negative effect on inter-observer agreement (concordance correlation coefficient, 0.80) and a positive effect on the identification of the more abnormal limb (kappa value 0.56). These findings were unexpected. They may represent perceived differences due to small sample size rather than a real relationship. It might be that less experienced observers have difficulty interpreting gait changes on a videotape or simply that 3-dimensional live OGA (i.e. OGA without videotape) is superior to videotaped 2-dimensional OGA. In contrast, having the two observers evaluate the same video rather than evaluating the patient at different times during the same visit may increase agreement on which limb is more affected (or appears to be more affected). Comparison of live OGA and videotaped OGA inevitably leads to comparison of two different protocols. In the live OGA, observers were allowed to keep watching each dog walk until certain of the score, so that they were not hindered by the times during which the patient was trying to sit down, pulling at the leash, etc. In the videotaped OGA, the support staff continued videotaping the dog until good footage was achieved, in which the dog ambulated in a straight line at a consistent walking speed (i.e. discarding video in which the dog was refusing to move, etc.). The observers were then permitted to watch each video as many times as required to be certain of the score. These two methods were therefore inherently different which could have created differing levels of agreement between the two observers. For example, a patient repeatedly collapsing on the same pelvic limb during turning would have been apparent to the observers via live OGA but unknown to the observers with videotaped OGA. On the other hand, the relatively symmetrically affected patient making similar numbers of mistakes with the left and right pelvic limbs could result in apparent agreement upon the worse limb by analysis of the same video but disagreement with live OGA. Clearly, the comparison of live versus videotaped OGA needs further study.

In an OGA scoring system, observers need to be trained before implementation because there is a learning curve associated with the scoring system. The goal of the training phase was to allow the observers to be familiar with the scoring system, and to similarly interpret the definitions of each grade of dysfunction. One potential limiting factor in scoring limbs individually for movement is the interconnected nature of the quadruped gait [56]. That is to say, a neurological dysfunction of the left pelvic limb will affect how the right pelvic limbs is used, and vice versa. In our study, a training period involving five dogs was carried out. Potentially, with poor agreement between the two investigators, a longer training period would have been beneficial. However, as most disagreements were by just one point, and failure to agree on the more abnormal limb only occurred with relatively symmetrical dysfunction, it is possible that further training might have only moderately increased agreement. Similarly, the use of only two observers with similar clinical backgrounds may have impacted the agreement.

The limitations of this study include the small sample size. This was partly due to the inclusion criteria, in which non-ambulatory patients were excluded, significantly reducing the number of myelopathy patients who were eligible. The potential impact of small sample size when evaluating inter-rater agreement is that disagreement on one patient could have a large impact on the kappa value. It is important to note that there was no randomization of patients to the two groups, which when combined with the small sample size means that the observed differences when using a videotape could be due to many other factors besides videotaping. This was a preliminary assessment of two different ways of implementing a new scoring system, and further study is required. While we have modified a prior OGA method [13] for scoring ambulatory canine myelopathy patients so that it might be of particular use to compare to IGA findings, an additional limitation is that our scoring system is not appropriate for non-ambulatory patients. Additionally, we included all types of myelopathy patients, and the ideal scoring system for patients with acute spinal cord injury may be different to the optimum scoring system for chronic diseases.

Future studies should further optimize the scoring system for OGA and investigate the significance of performing live OGA or videotaped OGA. Studies are on-going in these avenues, and in investigating the relationship and utilities of videotaped OGA and IGA.

## Conclusions

The modified scoring system in this study yields a separate functional score for each of the two pelvic limbs in ambulatory dogs with T3-L3 myelopathies. There was moderate reliability between two observers in individually assessing the functional neurologic status without videotape. Performing observations by reviewing videotapes might help experienced observers to acquire more precise evaluation of minor differences, or might allow more repeatable implementation of the scoring system by reviewing videos in batches. The modified functional scoring system is easily administrated clinically and required minimal training. This is the first OGA system to provide a separate score for each pelvic limb while recognizing many levels of ambulatory paraparesis-ataxia, which may provide a better ability to compare OGA with IGA in T3-L3 myelopathy dogs.

## Abbreviations

IGA: Instrumented gait analysis
OGA: Observational gait analysis
